# The SwissFEL soft X-ray free-electron laser beamline: Athos^1^

**DOI:** 10.1107/S1600577519003928

**Published:** 2019-06-21

**Authors:** Rafael Abela, Arturo Alarcon, Jürgen Alex, Christopher Arrell, Vladimir Arsov, Simona Bettoni, Markus Bopp, Christoph Bostedt, Hans-Heinrich Braun, Marco Calvi, Tine Celcer, Paolo Craievich, Andreas Dax, Philipp Dijkstal, Sladana Dordevic, Eugenio Ferrari, Uwe Flechsig, Rolf Follath, Franziska Frei, Nazareno Gaiffi, Zheqiao Geng, Christopher Gough, Nicole Hiller, Stephan Hunziker, Martin Huppert, Rasmus Ischebeck, Haimo Jöhri, Pavle Juranic, Roger Kalt, Maik Kaiser, Boris Keil, Christoph Kittel, René Künzi, Thomas Lippuner, Florian Löhl, Fabio Marcellini, Goran Marinkovic, Cigdem Ozkan Loch, Gian Luca Orlandi, Bruce Patterson, Claude Pradervand, Martin Paraliev, Marco Pedrozzi, Eduard Prat, Predrag Ranitovic, Sven Reiche, Colette Rosenberg, Stephane Sanfilippo, Thomas Schietinger, Thomas Schmidt, Kirsten Schnorr, Cristian Svetina, Alexandre Trisorio, Carlo Vicario, Didier Voulot, Ulrich Wagner, Hans Jakob Wörner, Adriano Zandonella, Luc Patthey, Romain Ganter

**Affiliations:** a Paul Scherrer Institut, CH-5232 Villigen, Switzerland; b École Polytechnique Fédérale de Lausanne (EPFL), CH-1015 Lausanne, Switzerland; c ETH Zürich, Zürich, Switzerland

**Keywords:** FEL, superradiance, APPLE undulator, chicane

## Abstract

The SwissFEL soft X-ray free-electron laser (FEL) beamline Athos will be ready for user operation in 2021. Its design includes a novel layout of alternating magnetic chicanes and short undulator segments. Together with the APPLE X architecture of undulators, the Athos branch can be operated in different modes producing FEL beams with unique characteristics ranging from attosecond pulse length to high-power modes. Further space has been reserved for upgrades including modulators and an external seeding laser for better timing control. All of these schemes rely on state-of-the-art technologies described in this overview. The optical transport line distributing the FEL beam to the experimental stations was designed with the whole range of beam parameters in mind. Currently two experimental stations, one for condensed matter and quantum materials research and a second one for atomic, molecular and optical physics, chemical sciences and ultrafast single-particle imaging, are being laid out such that they can profit from the unique soft X-ray pulses produced in the Athos branch in an optimal way.

## Abstract 2

An overview is given of the SwissFEL soft X-ray free-electron laser (FEL) beamline, called Athos, and its numerous operation modes. In particular, several key hardware components are described, which enable these modes. The main FEL parameters and expected performance figures are also reported.

## 1. Introduction   

Functional molecules and materials are of vital importance in today’s world. They can be part of catalytic systems to produce plastics or purify gases or synthesize fuels, ultrafast electronic switches and high-capacity magnetic storage media in information technology, or molecular complexes which govern cellular function and could cause hereditary disease. The cogs in such functional molecules and materials are the valence electrons, which, due to their electric charge, light mass and moderate binding energy, determine the physical, chemical and biological properties of matter. With soft X-ray spectroscopy, however, one can use well-defined resonant atomic transitions to specifically address particular electron orbitals. For this reason, a variety of soft X-ray spectroscopy methods are now ‘bread and butter’ for users of many synchrotrons in operation worldwide. In order to study and quantify the functionality of matter, it is necessary to follow the dynamics of valence electrons on their natural time scale. Only a soft X-ray free-electron laser (FEL), such as Athos at SwissFEL, offers the combination of peak intensity, wavelength tunability and femtosecond pulse length required to perform dynamic soft X-ray spectroscopy.

**Figure 1:**
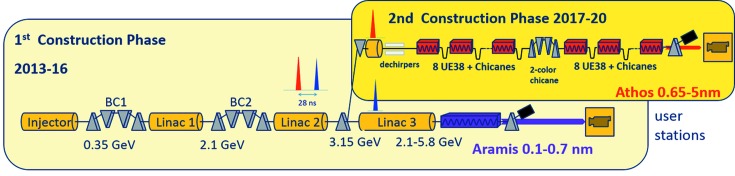
Schematic layout of SwissFEL with the Athos branch in the upper right part.

The hard X-ray FEL line of SwissFEL (Abela *et al.*, 2012 ▸; Milne *et al.*, 2017 ▸), Aramis (Fig. 1 ▸), has provided photon pulses to users since the end of 2017. With a nominal electron beam energy of 5.8 GeV, the Aramis FEL line covers a photon energy range from 2.0 to 12.4 keV. The soft X-ray range, from 0.25 to 1.9 keV, will be covered by another FEL line, Athos (Abela *et al.*, 2017 ▸). The layout of the Athos FEL line differs from most existing FELs, because it incorporates small magnetic chicanes after every undulator segment. With these chicanes the electron bunch can be manipulated (delayed, shifted horizontally, compressed) to optimize the lasing process and improve specific properties of the FEL light (spectral bandwidth, peak power, duration *etc*.). A new undulator geometry, APPLE X (Calvi *et al.*, 2017 ▸; Schmidt & Calvi, 2018 ▸), has been developed for Athos, in which a transverse field gradient as well as full control of the polarization can be obtained. The combination of these technical innovations opens the door to many different modes of operation providing a large variety of FEL properties.

To enable the parallel operation of Aramis and Athos at 100 Hz repetition rate, two bunches, separated by 28 ns but in the same radiofrequency (RF) macropulse, are accelerated up to a fast kicker, which will deflect one bunch into the Athos dogleg at an energy of 3.15 GeV (Fig. 1 ▸) while the other bunch goes straight to the Aramis beamline. The commissioning of the Athos dogleg has started already, demonstrating the operation of the fast kickers and the acceleration of two bunches in the injector. To facilitate the setup of the different FEL schemes, a transverse deflecting cavity situated downstream of the undulator will be installed as a diagnostics tool.

The optical layout will ultimately serve three experimental stations, but only the atomic, molecular and optical station (AMO) and the condensed matter station (CM or Furka) are currently under design. The commissioning of the undulator line should be completed in 2020 with user operation to follow in 2021.

## 2. Operation modes of Athos   

The design of the Athos beamline aims to go beyond the standard self-amplified spontaneous emission (SASE) operation to offer novel modes of operation to the users (Abela *et al.*, 2017 ▸). One unique feature is the radially movable magnetic arrays of the undulator modules, referred to as the APPLE X configuration (Schmidt & Calvi, 2018 ▸). The design allows tuning of the *K* value without shifting the magnet arrays longitudinally, that is to say without changing polarization. When the four magnet arrays are positioned in an asymmetric way with different left and right gaps, then a transverse field gradient can be generated for all polarizations: the so-called transverse-gradient undulator (TGU) configuration (Calvi *et al.*, 2017 ▸).

The module length has been optimized to increase the performance of advanced modes (Prat, Calvi, Ganter *et al.*, 2016 ▸) and, at 2 m, is shorter than needed for standard SASE operation. In addition, permanent magnet chicanes are placed between the undulator modules, which can delay the beam by up to 5 fs per break section with respect to the radiation field to improve radiation power and temporal coherence. Later, we refer to them as CHIC (chicanes for high power and improved coherence). In the baseline design, there is about 35 m available space in front of the undulator, which can hold possible implementations of laser–electron beam interaction schemes or spectral filtering with self-seeding (Feldhaus *et al.*, 1997 ▸; Saldin *et al.*, 2001 ▸; Geloni & Saldin, 2010 ▸; Amann *et al.*, 2012 ▸; Ratner *et al.*, 2015 ▸).

Beyond the standard SASE operation modes, such as femtosecond pulses with emittance spoiler slits (Emma *et al.*, 2004 ▸) or tilted beams (Prat *et al.*, 2017 ▸), two-colour operation (Reiche & Prat, 2016 ▸; Lutman *et al.*, 2016 ▸) and harmonic lasing (Schneidmiller *et al.*, 2017 ▸; Schneidmiller & Yurkov, 2012 ▸), there are four hardware modifications that open up new modes. Some of them already exist at other facilities, while others are truly unique to the Athos beamline. The four additional features of the undulator beamline are:

(i) The operation of the APPLE X undulator with a *transverse gradient* for all polarizations implemented with asymmetric radial distances of the four magnetic arrays.

(ii) The utilization of small delaying *chicanes* between all undulator modules.

(iii) The manipulation of the electron beam with an *external laser* before the injection into the main undulator beamline.

(iv) The spectral filtering of the SASE signal in between the FEL amplification process by means of a high-resolving *monochromator* (not in the baseline design).

**Figure 2:**
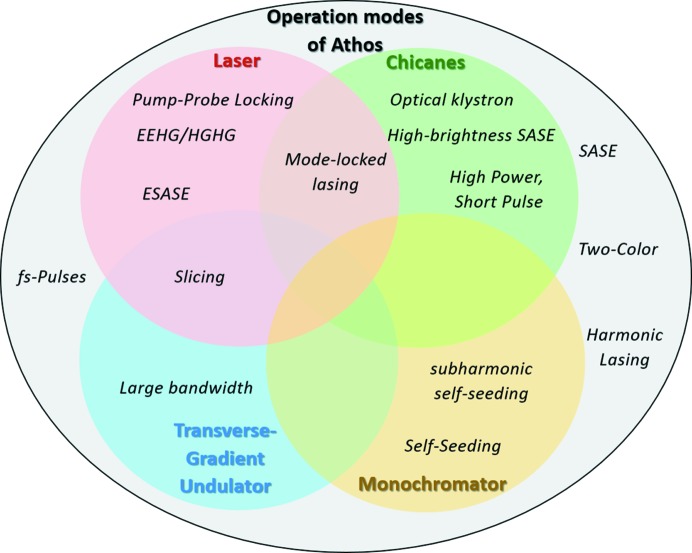
Possible operation modes of Athos, arranged by their required hardware components.

The interplay of these modes gives unprecedented control of the spectral coherence, pulse length and radiation power independently. The schematic view in Fig. 2 ▸ lists all possible operation modes which have so far been studied for Athos. Additional modes are currently being explored. In the following, brief explanations of these advanced modes are given (see also Table 1 ▸).

### 2.1. Transverse-gradient undulator configuration   

In the TGU configuration the undulator field strength has a linear dependence on transverse position. The strength and direction of the gradient can be controlled with the position of the four magnet arrays. For Athos, this unique undulator field property allows for two modes. The first is a large-bandwidth mode, where a spatially tilted beam is injected into the undulator lattice without any external focusing (Prat, Calvi & Reiche, 2016 ▸). Note that the natural focusing is present but the overall phase advance in the betatron oscillation is practically insignificant at the typical Athos beam energies of around 3 GeV. Since each part of the electron beam propagates parallel to the undulator axis, the FEL amplification, and thus the resonant wavelength, is defined by the local field strength, which varies due to the TGU configuration. Therefore the resulting FEL pulse has a spatial frequency chirp, whose magnitude can be controlled by the amplitude of the tilt and the field gradient. In contrast to other large-bandwidth modes, which are achieved with strong wakefield effects (such as occurring by overcompression of the electron beam in the last compression stage), in this method the direction of the frequency chirp (either low or high photon energies first) can be controlled. The drawback is the loss of focusing and thus weaker FEL performance. Simulation studies have shown that the chirp can be up to 20%.

The other possibility with the TGU configuration consists of applying a linear undulator taper within each module by tilting the module around its yaw axis. In this way, the transverse gradient is converted into a longitudinal gradient from the perspective of the electron orbit. This possibility of very fine taper is needed for electron slicing (see below) since a step-wise taper per undulator segment is not efficient enough.

### 2.2. CHIC modes using inter-undulator magnetic chicanes   

The most important difference of Athos in comparison with other FEL beamlines is the inclusion of small delaying chicanes between each undulator segment. One of its key benefits is not directly visible to the user, namely the reduction of the saturation length by a distributed optical klystron (Vinokurov & Skrinsky, 1977 ▸; Brautti *et al.*, 1978 ▸; Coisson, 1981 ▸) configuration. Here the conversion of energy modulation by the FEL process into coherent bunching is enhanced by the dispersion of the chicanes. The limiting factor is the intrinsic energy spread which would damp the bunching, similar to Landau damping when the applied dispersion is too large. At optimum configuration the reduction in saturation length can amount to 20–30%. Another effect is that the chicanes spread the radiation field over the bunch faster than slippage alone, which accounts for one radiation wavelength per undulator period. As a result, the longitudinal coherence is improved and the SASE bandwidth narrowed. This is known as the high-brightness SASE (HB-SASE) mode (McNeil *et al.*, 2013 ▸).

Since the Athos chicanes offer both dispersion and delay, the two effects may be combined into a compact HB-SASE mode: while the saturation length is reduced, a careful tuning of the chicanes improves the spectral brightness. Simulations have shown that the increase in brightness is about a factor of ten at higher photon energies (above 1.24 keV) (Prat, Calvi, Ganter *et al.*, 2016 ▸) and the enhancement is still a factor three at the lower photon energy limit of Athos. Because self-seeding (see above) is not realized as a baseline option for Athos, this mode is in high demand by the users to increase the photon flux going through the monochromator. The main drawback is that the central frequency fluctuates with the energy jitter of the electron beam, in contrast to self-seeding where it is the central frequency that is stable while the output pulse energy jitters. Since it is straightforward to switch back to SASE by setting the chicanes to zero, the spectral bandwidth can be adjusted to the individual needs and requirements of the users.

Another unique scheme offered by the delaying chicanes is the high-power short-pulse mode, where a short radiation pulse can be shifted to a fresh, unspoiled part of the electron bunch, as long as the pulse is shorter than the delay (Prat & Reiche, 2015 ▸; Prat * et al.*, 2015 ▸). This can be used to exploit the superradiant (Bonifacio *et al.*, 1990 ▸, 1991 ▸) regime of an FEL. In the conventional case the amplification stops after SASE saturation occurs when the radiaton spikes slip into the region where the preceding spike has already spoiled the electron beam quality. If that part is unspoiled, however, then the spike can grow further, beyond the SASE saturation power level. The peak power grows quadratically with the distance in the superradiant regime, while the pulse length narrows with the inverse of the square root of the distance. In total, the pulse energy grows to very high values, ideal for user experiments studying non-linear dynamics. In Athos, keeping part of the bunch unspoiled is achieved by injecting a tilted beam. Only the beam part aligned to the undulator axis will lase as the betatron oscillation suppresses the lasing amplification in the rest of the bunch. Once the initially lasing bunch slice is exhausted (*i.e.* its energy spread has become too large), the beam is delayed and realigned with the undulator axis so that a new fresh slice will continue the FEL amplification. While this principle has been demonstrated at LCLS (Lutman *et al.*, 2018 ▸) with two chicanes, at Athos it can be applied with many more chicanes, further sustaining the superradiant growth. Simulations at 2 nm have shown peak fields in excess of 1 TW with a pulse energy of 1 mJ and a pulse duration of 500 as (Prat, Calvi, Ganter *et al.*, 2016 ▸). For a radiation wavelength of 1 nm, the pulse energy would be 300 µJ with a pulse duration of 250 as.

### 2.3. Manipulation with external laser   

Upstream of the main undulator beamline the electron beam can be overlapped with an external laser, inside a dedicated undulator, also called a modulator. As a result, the bunch energy profile is modulated with the periodicity of the external laser wavelength. The first consequence is an energy modulation, but a small chicane prior to the main undulator line can convert this energy modulation into a current modulation.

The mode-locked lasing (Thompson & McNeil, 2008 ▸) requires a periodic modulation of the electron beam energy, as described above. For undulator segments, whose lengths are shorter than the gain length, a periodic delay introduced by the inter-undulator chicanes generates a modal structure in time and spectrum, which can be locked when a periodic perturbation is applied. This perturbation can be realized with the aforementioned energy or current modulation obtained with a laser. If the slippage within one module and the chicane delay match the periodicity of the perturbation, mode-locking occurs, where the modes are locked in phase. Indeed, the artificially increased slippage due to chicanes, which is identical at each chicane, will lead to the growth of regularly spaced spikes in the intensity time profile. These spikes or modes are locked to each other as well as to the external laser. In contrast to SASE spikes, the widths of these spikes can be shorter, according to the slippage within one module (*e.g.* at 2 nm for Athos the slippage length is 104 nm, corresponding to 350 as full width).

Enhanced SASE (ESASE) (Zholents, 2005 ▸) is similar to mode-locked lasing: it induces a current modulation but there is no locking of the pulses by the chicanes. Although each mode has its own phase, the power profiles or power spectra on average exhibit the same modal structure. It is easier to set up than mode-locked lasing and can be considered a pre­requisite study for the more advanced mode. The ESASE concept has not been demonstrated yet, neither in the soft nor in the hard X-ray regime.

The combination of an external laser (to modulate the bunch in energy) and a linear tapering (tapering within the undulator segment thanks to the TGU configuration) opens the door to ‘slicing’ techniques (Saldin *et al.*, 2006 ▸). In the ‘slicing’ scheme, a very strong energy modulation is combined with a strong taper. In the rising part of the energy modulation, the FEL interaction is preserved since the slippage of the radiation field into parts of the electron bunch with higher beam energies is kept in resonance with the growing undulator field. In the other part of the bunch, on the falling flank of the modulation, lower beam energies but higher undulator fields disrupt the FEL process. The energy modulation can either be done with a single-cycle carrier-phase-envelope stabilized laser pulse or – less challenging – with a long laser pulse but using an emittance spoiler foil, which corresponds to the XLEAP setup at LCLS (MacArthur *et al.*, 2017 ▸). The result of these slicing methods is a short (attoseconds long) FEL pulse, which is locked to the radiation phase of the external laser field.

Triggered by recent progress at the FERMI light source, the echo-enabled harmonic generation (EEHG) (Stupakov, 2009 ▸; Zhao *et al.*, 2012 ▸) method was re-evaluated for SwissFEL. While it seems impractical for covering the full wavelength range, given the excessive size of the first chicane and the degrading effects of intra-beam scattering and quantum fluctuation of the incoherent synchrotron radiation, it is feasible for lower photon energies up to 500 eV. As such, EEHG is complementary to HB-SASE, since HB-SASE works best for higher photon energies.

A multi-stage, fresh-bunch approach of high-gain harmonic generation (HGHG) (Yu, 1991 ▸) is not suitable for Athos without a major change in the layout, which would be mutually exclusive with other modes. However, the similar concept of coherent-harmonic generation (Girard *et al.*, 1984 ▸) might be possible, at the expense of lower pulse energies. This method is currently being investigated for the specification of a possible intermediate undulator line. This undulator line would be the same as that used to drive a possible self-seeding setup.

All laser manipulations affecting only a subsection of the bunch offer the additional benefit of an FEL signal that is passively locked to an optical laser. If both signals can be transported in parallel down to the user station, the jitter of pump–probe experiments can be reduced significantly. This has been demonstrated at FERMI with relative fluctuations of 6–9 fs (Cinquegrana *et al.*, 2014 ▸).

### 2.4. Self-seeding configuration   

The most direct benefit of a self-seeding configuration is the reduction of the FEL bandwidth with significantly improved temporal coherence. There is not enough space in one undulator section (2 m length) to install a monochromator as was done at LCLS (Ratner *et al.*, 2015 ▸). Therefore, the idea is to place, in the available space in front of the baseline undulator line, the filtering monochromator, a delaying chicane and some additional undulator modules to provide a sufficiently strong SASE signal. The allocated space for the chicane is 10 m and the electron beam itself acts as a kind of ‘pinhole’ to pick up the desired central wavelength. In this approach, the resolving power can be increased more than 10 000-fold.

The monochromator also offers the possibility to make use of harmonic lasing (Schneidmiller & Yurkov, 2012 ▸; Schneidmiller *et al.*, 2017 ▸). Instead of operating the monochromator at the final wavelength, it can be tuned to a subharmonic wavelength (Geloni *et al.*, 2011 ▸, 2015 ▸; Prat & Reiche, 2018 ▸). The first part of the radiator is tuned to the subharmonic until the coherence properties due to the non-linear terms are transferred to the final wavelength. This offers two advantages. The first is that with this harmonic conversion the effective resolving power of the monochromator is enhanced. For example, a resolving power of 10 000 at 1 nm introduces a coherence length of about 10 µm, while at 3 nm it is 30 µm. Since the coherence properties are transferred to the harmonics, this harmonic conversion leads to an increase of the coherence length. The second advantage is due to the fact that part of the undulator line is operated at the subharmonic wavelength with a shorter gain length, leading to an overall more compact setup. In simulation we see a reduction in the bandwidth by a factor of two while requiring between four and six fewer undulator modules compared with direct self-seeding.

## 3. Main electron beam components   

Two distinct laser systems will be used to extract two bunches from the photocathode with 28 ns separation. This will provide individual control of the repetition rate of each FEL up to a maximum of 100 Hz for both FELs. The two bunches are always in the same RF macropulse during the acceleration from the injector to the end of linac 2, posing the difficulty of individual control of the RF phase for each bunch. Preliminary tests in the SwissFEL injector have demonstrated the feasibility of two-bunch transport. Further preparation work is underway to allow control of the phase of the second bunch independently of the first bunch within some limits. In the C-band accelerator of linac-1 and -2, the compressed RF pulses must be shifted in time to provide the same energy gain to both bunches. In the switchyard, the two bunches are separated by the kicker system described in the following.

### 3.1. Kicker and septum (Paraliev *et al.*, 2014 ▸; Paraliev & Gough, 2017 ▸)   

**Figure 3:**
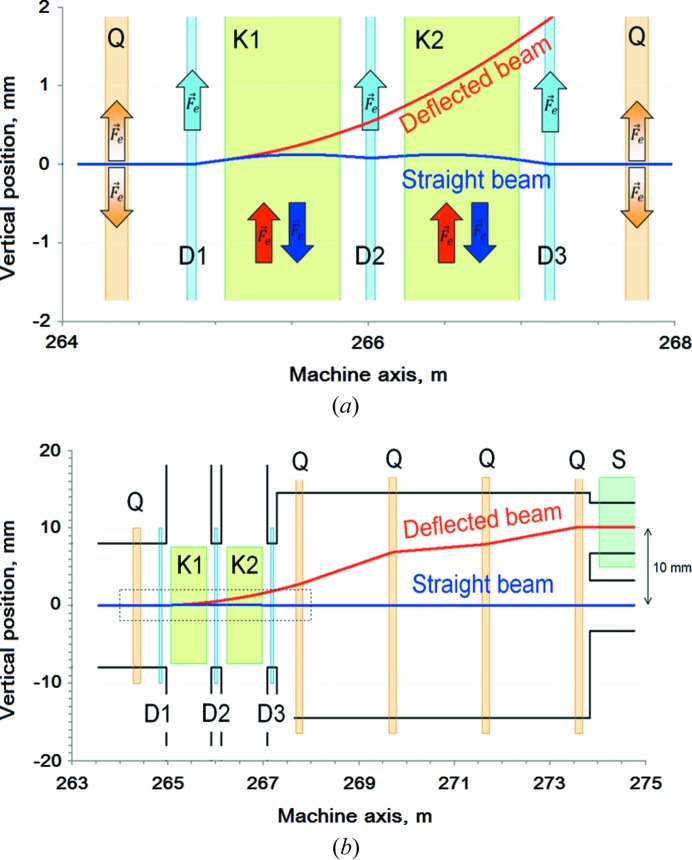
Vertical plane beam trajectories between kicker and septum (*a*) and the same but magnified in the kicker region (*b*). Coloured rectangles represent the field regions of different magnetic components: quadrupoles (Q), kickers (K), dipoles (D) and septum (S). Arrows show the direction of magnetic deflection.

Separation of the two closely spaced bunches is achieved using two resonant deflecting magnets (kickers), three compensating dipoles and a septum magnet, shown in Fig. 3 ▸.

The two kicker magnets (K1 and K2) separate the bunches vertically by ‘kicking’ one bunch up and the other one down. Compensating dipoles (D1, D2 and D3) bring the down-kicked bunch back to the straight-through machine axis and deflect further up the up-kicked one. The septum is positioned 8 m downstream of the separation point. The deflected bunch enters the septum dipole field with 10 mm separation from the straight-through bunch and is deflected 35 mrad towards the Athos beamline; the lower beam passes through the zero-field region of the septum to continue straight on to the Aramis beamline (see Table 2 ▸).

#### 3.1.1. Resonant kickers   

Resonant deflection is used to give moderate field strength but very stable operation. An RF driver excites a sine-wave current in the high-*Q* lumped resonator. The current is precisely synchronized to a common subharmonic of the SwissFEL RF operating frequencies, namely 17.85 MHz; this gives 28 ns per half-cycle. Once the resonating current (and hence magnetic field) is established in the kicker, electron bunches can be deflected alternately up and down. The kickers’ resonators, coaxial cables and electronics require stable water cooling.

#### 3.1.2. Lambertson septum   

**Figure 4:**
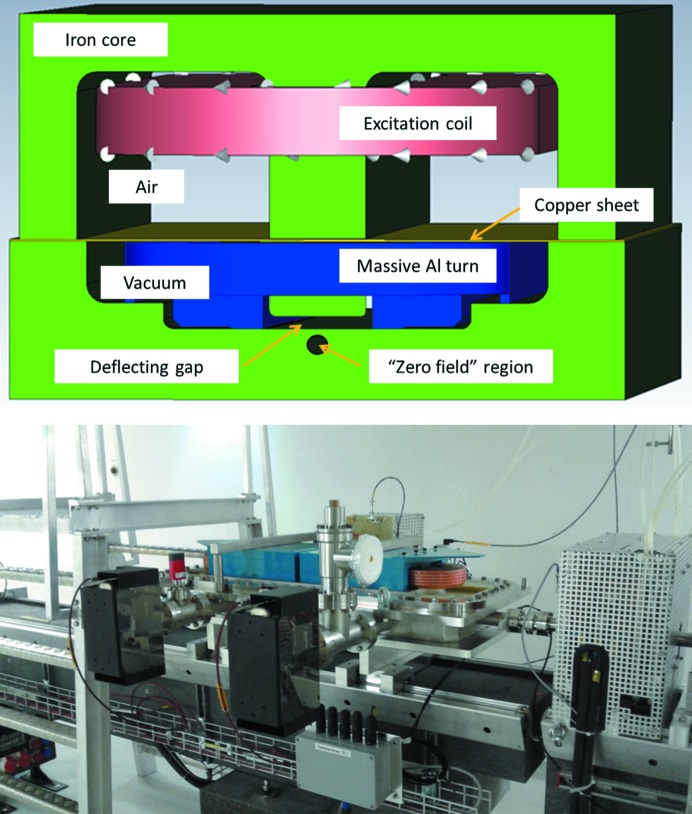
Schematic and photograph of the Lambertson septum.

The DC Lambertson septum magnet (see Fig. 4 ▸) is a ‘half-in-vacuum’ design with relatively small geometry for this FEL application. The vertical bunch separation is 10 mm and an 8 mm-diameter hole was drilled through the iron core for the straight-through path in the zero-field region. A 1 mm-thick copper sheet separates air from vacuum and the dipole magnetic field passes through this sheet. The excitation coil is in air and is water cooled. Eddy currents in a massive aluminium turn and in the iron core limit the transfer of power supply current noise above ∼0.1 Hz.

### 3.2. Dechirpers   

**Figure 5:**
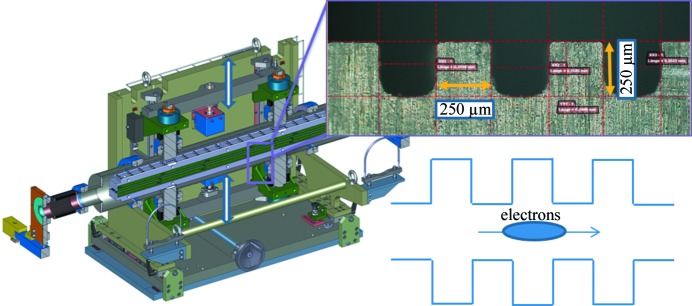
Dechirper vacuum chamber where two parallel corrugation plates (insets) can be moved close to the beam trajectory.

In recent years several possibilities have been considered to manipulate a relativistic beam using its interaction with the longitudinal and transverse wakefields excited by the bunch passing in a dielectric lined or corrugated waveguide. The dechirper is a device used to control wakefields by means of parallel plates with microstructured surfaces (Antipov *et al.*, 2014 ▸; Emma *et al.*, 2014 ▸). In the section between the switchyard separation point and the first Athos undulator, a sequence of eight flat corrugated plates as shown in Fig. 5 ▸ (Ganter *et al.*, 2017 ▸) will be installed. Two units of these dechirpers are currently being commissioned at SwissFEL. The excited longitudinal wakefield can be used to remove the residual energy correlation along the bunch resulting from the previous compression stages (dechirping the bunch). Dechirpers can also be used to tilt the bunch transversally such that only a portion of the bunch will be aligned with the undulator axis and meet lasing conditions as described in Section 2 for the special operation modes of Athos. Indeed, when the bunch enters the dechirper gap off axis it will be slowly tilted.

### 3.3. APPLE X undulators   

**Figure 6:**
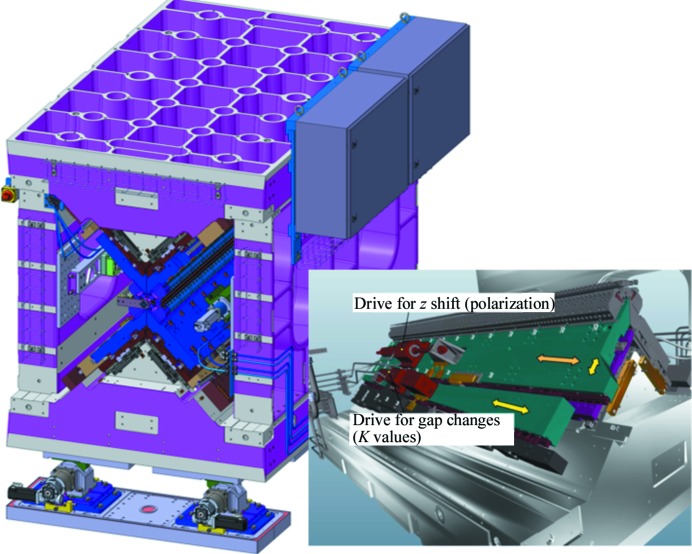
The UE38 undulator is about 2.2 m long, 1.4 m wide and 2 m high. The cast iron frame is adjustable with five degrees of freedom thanks to cam-shaft movers. The motor control electronics are mounted directly to the frame. Inset: magnet arrays can move along a 45° plane to vary the gap (*K* values) and along the *z* axis to change polarization.

For the Athos beamline a new APPLE-type undulator concept (Fig. 6 ▸) has been developed to provide all polarization modes over the entire wavelength range (Schmidt & Calvi, 2018 ▸). The four magnet arrays are individually adjustable in the radial direction, at an angle of 45° relative to the vertical plane, as well as in the axial direction. The photon energy and polarization of the emitted light can then be adjusted independently with the help of eight motors. The result is a fully symmetric design with equal vertical gaps and horizontal slits for all *K* values. In addition, the mechanics allow operation in an asymmetric way with left and right arrays at different gaps to generate a transverse magnetic field gradient. Details on the operation with transverse gradients in all polarization modes for the APPLE X can be found in the work of Calvi *et al.* (2017 ▸).

The mechanical support is a closed frame with two stiff triangular base modules carrying the drive systems under a 45° angle and two side walls made of cast iron as shown in Fig. 6 ▸. The radial plates moving along the 45° plane can be adjusted by ±7.5 mm using a wedge system sufficient to vary the *K* value between 1 and 3.8. The upper plate on the triangular base moves along the beam axis directly with a stepper motor to adjust the polarization.

The individual magnet geometry follows from the symmetry of the APPLE X topology. The undulator period is 38 mm with samarium cobalt (SmCo) magnets. Two different grades of SmCo materials are used: Sm_2_Co_17_ for the radially magnetized magnets and SmCo_5_ for the axially magnetized magnets. The magnets are combined accordingly in a Hallbach structure. SmCo magnets are in general less strong compared with the widely used neodymium ones (NdFeB), but they are two to three times less sensitive to temperature fluctuations. In addition, the permeability of the axial magnet is closer to unity, thus reducing non-linearity (hysteresis) effects when shifting the magnet arrays. Radial magnets have been magnetized under an inhomogeneous field such that the field is larger near the apex of the magnet while reducing the overall magnetic load. The magnetic field measurements of the individual magnet pairs (radial and axial) confirmed that they meet the quality required for undulators.

**Figure 7:**
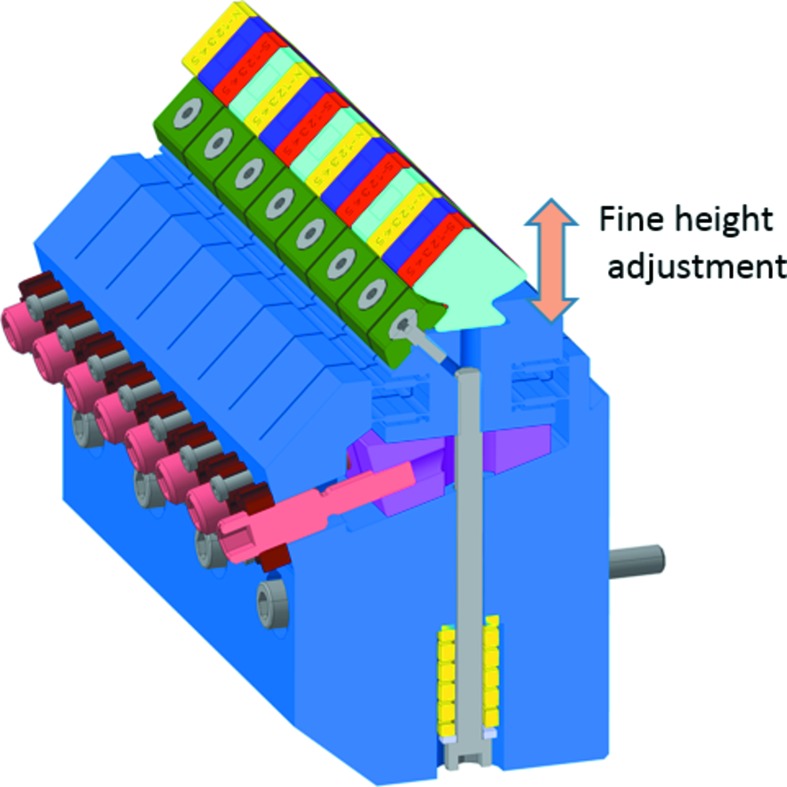
Block keeper principle with two magnets per individual flexor and four periods in total.

Four periods (16 magnets) are positioned in a block keeper as shown in Fig. 7 ▸. Every pair of magnets can be fine-adjusted in position thanks to a spring-loaded flexor system. Such a keeper allows an automated shimming to adjust the field profile as is done for the in-vacuum U15 planar undulators of the SwissFEL Aramis beamline.

When the gap and slit are at minimum, an aperture of only 6.5 mm is left open for the vacuum chamber. For this reason, a round copper vacuum chamber with 5 mm inner diameter and a wall thickness of only 0.2 mm was chosen. A vacuum level of around 10^−8^ mbar is expected with ion getter pumps situated in the inter-undulator sections. The assembly of the prototype undulator UE38 was completed in 2018.

### 3.4. X-band post-undulator transverse deflecting cavities   

**Figure 8:**
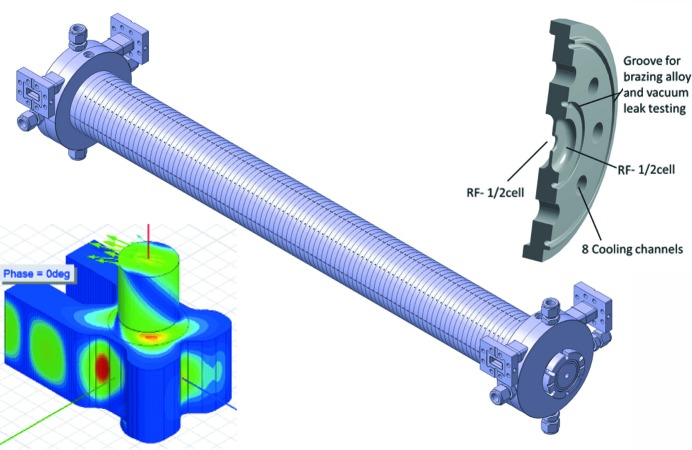
Left: detail of the RF field in the input/output coupler. Middle: complete TDS prototype. Right: basic disc.

Electron beam diagnostics based on a transverse deflection structure (TDS) placed downstream of the undulators (post-undulator TDS) in conjunction with an electron beam energy spectrometer can indirectly measure the pulse duration of ultrashort FEL pulses by analysing the induced energy spread on the electron bunch due to the FEL process (Dolgashev, 2014 ▸; Behrens *et al.*, 2014 ▸). Furthermore, a complete characterization of the electron beam 6D phase space by means of measurements of bunch duration, energy spread and transverse slice emittances (vertical and horizontal) are important tasks for commissioning and optimization of the FEL (Alesini *et al.*, 2006 ▸; Akre *et al.*, 2001 ▸; Ego *et al.*, 2015 ▸; Craievich *et al.*, 2015 ▸). In this context, the design of a new X-band TDS, namely the polarizable X-band (PolariX) TDS, was proposed by CERN (Grudiev, 2017 ▸). To avoid the rotation of the polarization of the dipole fields along the structure, a high-precision tuning-free assembly procedure, developed for the C-band linac at the Paul Scherrer Institut (PSI), will be used for the fabrication of the TDSs (Ellenberger *et al.*, 2013 ▸). Several facilities at DESY (FLASH2, FLASHForward, SINBAD) are also interested in the utilization of high-gradient X-band TDS systems for high-resolution longitudinal diagnostics. In this context a collaboration between DESY, PSI and CERN was established with the aim of developing and building an advanced X-band TDS with the new feature of variable polarization of the deflecting field (Craievich *et al.*, 2018 ▸). Bead-pull RF measurements were performed at PSI in 2018 to verify that the polarization of the dipole fields does not exhibit any rotation along the structure. Fig. 8 ▸ shows the details of the input and output couplers, the TDS prototype and the basic disc geometry.

**Figure 9:**
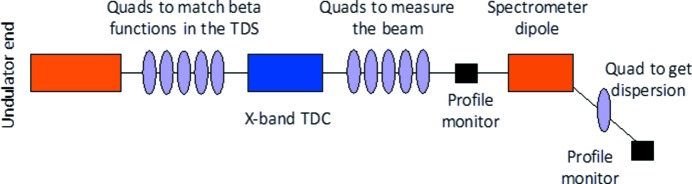
Concept of the post-undulator diagnostic section.

Fig. 9 ▸ shows a schematic layout of the post-undulator diagnostic section. Beam slice emittance in both transverse planes will be measured by a multi-quadrupole scan technique combined with the TDS. By means of the TDS, the beam is vertically or horizontally streaked and a multi-quadrupole scan is performed to determine the slice emittances (Prat & Aiba, 2014 ▸). For this purpose, it is envisaged that five quadrupoles will be placed downstream of the TDS. Reconstruction of the longitudinal phase space will be achieved by means of the spectrometer line. The time resolution is expected to be in the sub-femtosecond range when applying a deflecting voltage of 45 MV. Such time resolutions will allow the characterization of the shortest electron beam current profile foreseen for the Athos beamline with approximately two to three slices (Craievich *et al.*, 2017 ▸).

## 4. Optical layout   

The Athos beamline distributes the FEL radiation to three independent experimental stations with sufficient space in-between. A first horizontally deflecting mirror is located in the optics hutch at 54 m from the end of the undulator. It separates the FEL radiation from the *Bremsstrahlung* generated in the accelerator. Following the offset mirrors, the Athos 2 branch is designed to take the straight, pink beam while the Athos 1 and Athos 3 branches will use a monochromator. For the latter two, the FEL beam is deflected into a grating chamber with two interchangeable gratings and a plane mirror in an SX-700 (Riemer & Torge, 1983 ▸; Petersen, 1982 ▸) geometry, while the *Bremsstrahlung* is blocked after the first mirror. Additional shielding behind the grating reduces the radiation level further. The SX-700-like mechanics in the grating chamber introduce a vertical beam offset of 9 mm and change the transmitted photon energy by simple rotations of the plane mirror and the grating. After the grating chamber, the beam is inclined and points upward by 0.15°. This increases the reflectance on the plane mirror while keeping the included angle on the grating at a reasonable value. The beamline is equipped with two spherical gratings with variable line spacing and line densities of 50 and 150 lines mm^−1^, respectively. Both gratings can cover the full photon energy range of the beamline. The low-line-density grating provides moderate resolving power with short pulse stretching, while the high-line-density grating aims for high resolving power with an unavoidably larger pulse stretching. The gratings are of cylindrical shape with a radius of 7 km and are able to focus the zero order onto the exit slit. The capability to focus the zero order is essential for *in situ* wavelength calibration after a grating change. Furthermore, also in the Athos 1 and 3 branches a dedicated pink-beam mode can be employed by retracting the grating and using the beamline with two additional mirrors (*z* = 75 and 77 m), thus utilizing reflective optics only.

**Figure 10:**
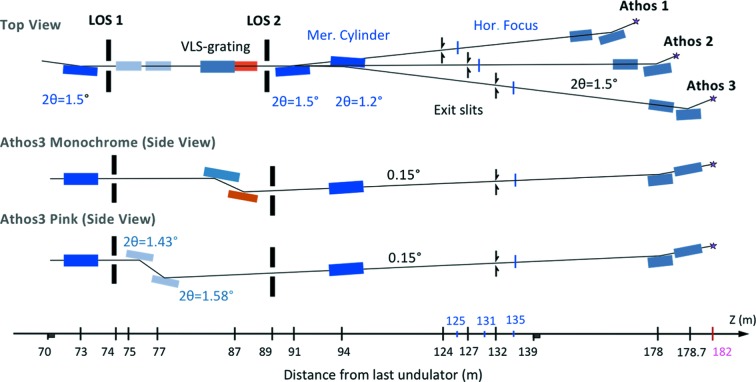
Optical design of the Athos beamlines.

After the grating chamber the beamline splits into three branches by introducing horizontally deflecting mirrors for Athos 1 and Athos 3 while Athos 2 is operated without such a mirror. Each branch has a separate exit slit and a dedicated Kirkpatrick–Baez (KB) mirror chamber for refocusing the beam into the experimental stations (see Fig. 10 ▸).

The optical elements in the FEL beamlines should consist of low-*Z* material or have at least low-*Z* coatings and operate under shallow incidence angles to avoid peak power damage. The Athos beamline uses silicon as bulk material for all optical elements. The advantage of silicon is the absence of absorption edges up to 1800 eV. For higher photon energies, a Rh/B4C bilayer is anticipated. Switching is done by a lateral translation of the elements. This does not affect their optical properties, as all elements are of cylindrical shape. The monochromator performance was assessed with ray tracing. An ultimate energy resolution of 10 meV at 250 eV is feasible with the high-line-density grating, increasing to 200 meV at 1800 eV.

## 5. Athos soft X-ray experimental stations   

**Figure 11:**
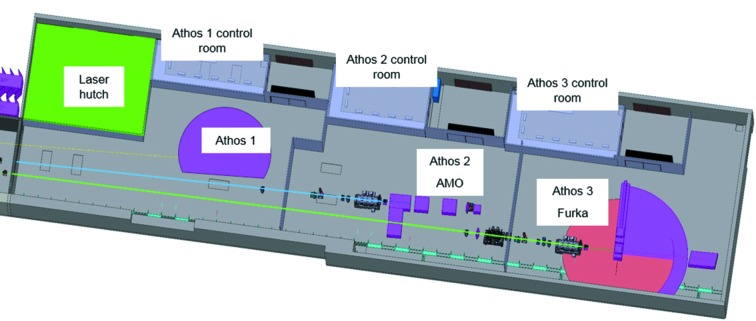
Sketch of the experimental areas of the SwissFEL Athos beamlines. The accelerator is to the left of the above area, with X-rays propagating from left to right.

The Athos soft X-ray experimental area, shown in Fig. 11 ▸, is contained within one large hutch with a floor space of 692 m^2^. Space within this hutch is allocated for the optical laser and the three experimental stations. Two experimental stations, one for condensed matter (Furka) and one for atomic, molecular and optical sciences (AMO), will be ready for the first experiments in 2021, while the third station (Athos 1 branch) is not yet defined and not yet financed either.

### 5.1. Experimental station for atomic, molecular and optical sciences   

The AMO experimental station at the Athos 2 branch will be designed as a highly versatile tool for AMO physics, chemical sciences, soft X-ray imaging, and novel approaches in non-linear X-ray spectroscopy. The X-ray pulses from the Athos undulator will be delivered with the minimum required three bounces from a single offset mirror and two KB mirrors. This optical layout will minimize transport losses and preserve the pulse wavefront. A short focal length of 1.5 m from the downstream KB mirror is chosen to achieve a micrometre-sized focus and therefore sufficient fluence for multi-photon excitations of the targets while maintaining enough space for laser incoupling elements and differential pumping between the last optical element and the experimental station.

A specific characteristic of the Athos AMO station will be the combination of an X-ray FEL with an infrared laser-driven attosecond high-harmonic generation (HHG) source. The combination of the two sources with their specific strengths will provide new tools for unravelling and controlling ultrafast chemical dynamics in gases, clusters and liquids from an entirely new point of view. In particular, electronic and nuclear dynamics could be measured in real time in isolated gas-phase molecules and clusters, solvated molecules, transition-metal complexes and nanoparticles in their natural environment. The femtosecond to sub-femtosecond intense X-ray pulses from the Athos undulator and the HHG source will be optimally exploited in combination with the element specificity of soft X-ray spectroscopy and the nanoscale resolution of X-ray imaging.

**Figure 12:**
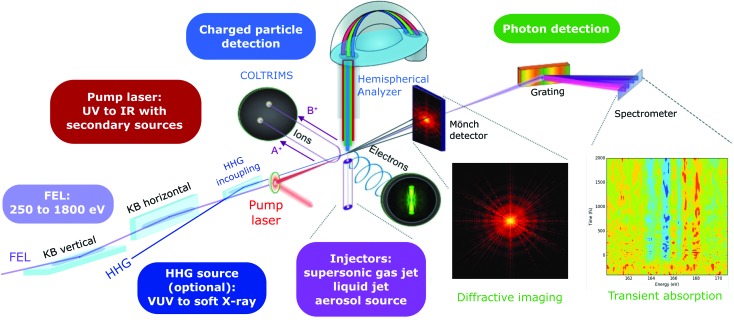
Conceptual design of the SwissFEL AMO beamline. The experimental station consists of the following sub-units: a main UHV chamber with electron and ion detection as well as a large-area X-ray imaging detector, and a transient absorption spectrometer. A variety of injectors will deliver cold atomic and molecular targets, liquid jets, as well as nanoparticles and aerosols.

Fig. 12 ▸ shows the conceptual design of the SwissFEL AMO experimental station. In the proposed station X-rays from the Athos undulator will be combined with a laser-driven atto­second VUV/XUV source based on HHG for a new class of pump–control–probe experiments, covering a broad photon energy range, ultrashort pulses and high-brilliance photon beams. Specifically, the ability to combine the HHG supercontinuum with the intense X-ray pulses from SwissFEL will allow us to translate transient absorption spectroscopy from the optical to the X-ray spectral domain and from valence to core and inner-shell orbitals. The narrow bandwidth and high photon flux of the Athos beamline will enable researchers to selectively ionize the core levels of specific elements, *e.g.* the *K* shells of carbon (290 eV), nitro­gen (410 eV), oxygen (540 eV) and fluorine (700 eV), but also the 3*d* transition-metal *L* shells of all elements between titanium (460 eV) and copper (950 eV). The transient absorption setup will be able to probe the X-ray-induced dynamics over the complete spectral range of the HHG supercontinuum in a single shot. Further, a variety of spectrometer and detector options, capable of detecting all reaction products, will be implemented, *i.e.* full 3D momenta of ions, high-resolution electron spectra, X-ray diffraction images and transient absorption spectra. Generally speaking, electron imaging spectrometers perform best for lower electron kinetic energies and are therefore not ideally suited for all X-ray energies. Therefore, we will complement the imaging spectrometers with a hemispherical analyser for electron detection. Another unique capability of the proposed experimental station will be the Mönch photon area detector, currently under development at the Paul Scherrer Institute, with dynamic gain switching. We aim at megapixel detectors but the exact pixel number and geometry will be the result of an optimization process of the Athos station, currently ongoing. A final and equally important aspect of the proposed station is sample handling and delivery. As sample delivery options at the AMO station we will provide supersonic jet sources for atomic, molecular and cluster beams. In addition, we aim at incorporating a flat liquid jet and an aerosol injector. The flat liquid jet will enable frontier experiments in chemical and biochemical research in the liquid phase, which is a particularly attractive combination with the X-ray-based transient absorption setup. The aerosol injector will allow us to extend the ultrafast imaging capabilities to a wide spectrum of chemical and biological systems.

In summary, the highly versatile AMO experimental station at SwissFEL will include a variety of particle and photon spectrometers, imaging capabilities and sample delivery systems. This station, in combination with ultrafast optical laser driven sources and the unique pulse characteristics from the Athos undulator, will enable new approaches in ultrafast and non-linear X-ray sciences.

### 5.2. Furka, the experimental station for condensed matter and quantum materials   

**Figure 13:**
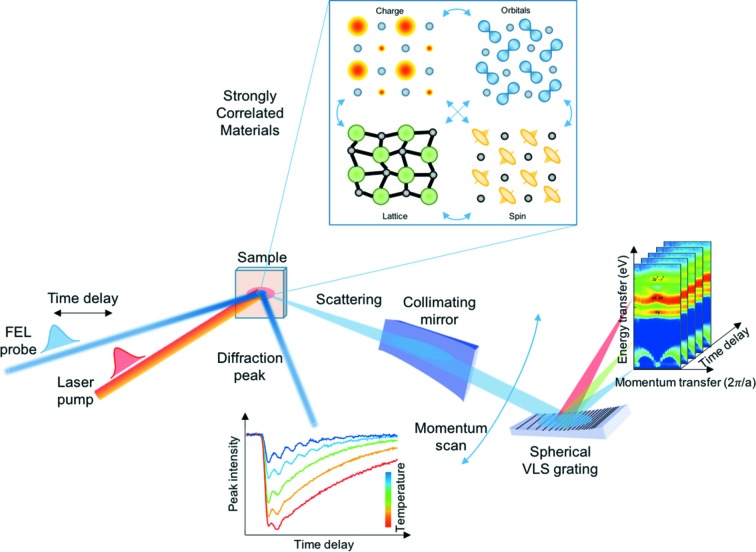
Schematic of the Furka experimental station performing ultrafast tr-RIXS, tr-REXS and tr-SXD on quantum materials [RIXS map adapted from Schlappa *et al.* (2012 ▸)].

The experimental station for condensed matter and quantum materials at the Athos beamline, named Furka, will be dedicated to time-resolved resonant inelastic and elastic X-ray scattering (tr-RIXS and tr-REXS) as well as soft X-ray diffraction (tr-SXD) to study ultrafast dynamics in correlated materials and, more generally, in quantum matter. Fig. 13 ▸ sketches the main features of Furka for both tr-RIXS, tr-REXS and tr-SXD. Many of the properties of quantum materials originate from couplings between charge, orbital, spin and lattice degrees of freedom. These couplings lead to cross-correlations among different physical observables, which develop towards the application of emergent functions (Tokura *et al.*, 2017 ▸). Mott transition, high-temperature superconductivity, topological superconductivity, colossal magnetoresistance, giant magneto-electric effect and topological insulators are just a few examples of remarkable functions and properties that arise from the collective behaviour of the different degrees of freedom. Ultrafast techniques, especially femtosecond spectroscopy or time-resolved X-ray diffraction, supported by the advent of Athos, now open new opportunities for direct measurements of the coupling strength between the different degrees of freedom at temperatures below 10 K, with unprecedented precision. In femtosecond pump–probe experiments, selective excitation is used to probe: (i) low-energy electronic, magnetic and structural dynamics; (ii) coupling and ordering dynamics of charge, orbital, spin and lattice in correlated systems; (iii) phase transitions and quasiparticle excitations away from equilibrium; (iv) correlations and fluctuations in non-equilibrium systems; (v) coupling, control and switching in quantum matter.

In the future, a second chamber is foreseen with the aim of investigating non-linear optical effects on solid materials as well as imaging techniques. Possible extensions to time-resolved X-ray magnetic circular dichroism (tr-XMCD) could be explored as well by taking advantage of the circular polarized radiation provided by Athos in combination with an externally applied magnetic field.

## Figures and Tables

**Table 1:** Key parameters for most of the operation modes discussed in this section Underlying simulations were based on 20 undulator modules available.

Mode	Pulse energy	No. of photons per pulse at 1 nm	Pulse duration (r.m.s.)	Bandwidth (r.m.s.)	Comment
SASE (200 pC)	>1 mJ	5 × 10^12^	30 fs	0.1–0.4%	
SASE (10 pC)	>50 µJ (10 pC)	2.5 × 10^11^	2 fs	0.1–0.4%	
Self-seeding	∼ 1 mJ	5 × 10^12^	30 fs	< 10^−4^	Above 1 nm, 200 pC only
Optical klystron	>1 mJ	5 × 10^12^	30 fs	0.1–0.4%	Shorter saturation length
HB-SASE	>1 mJ	5 × 10^12^	30 fs	0.01–0.13%	Can also be configured for pulse trains
High-power short-pulse	∼300 µJ	1.5 × 10^12^	∼250 as	1% FWHM	200 pC bunch
Two colours	2 × ∼50 µJ	2 × 2.5 × 10^11^	2 × 2–10 fs	0.2%; tuning range: factor 5	Based on 200 pC bunch
Large-bandwidth	∼0.1 mJ	5 × 10^11^	30 fs	>10% full width	200 pC only
Slicing	1 µJ (every 3 fs)	5 × 10^9^	< 1 fs per pulse	0.1–0.4%	Single pulse or pulse train; sub-femtosecond locking

**Table 2:** Main parameters of kickers and septum

Resonant kickers	
Number of devices	2
Deflection angle	±0.45 mrad
Frequency	17.85 MHz
Peak current	280 A
Peak current stability	<3 p.p.m. r.m.s.
Peak field	6.2 mT
Peak field integral	4.7 mT m
	
Septum	
Number of devices	1
Deflection angle	35 mrad
Current (MMF)	3700 At
Stability	<10 p.p.m. r.m.s.
Magnetic field	480 mT
Main field integral	365 mT m
Leakage field integral	<100 µT m
